# Transcriptome Landscape of *Mycobacterium smegmatis*

**DOI:** 10.3389/fmicb.2017.02505

**Published:** 2017-12-18

**Authors:** Xinfeng Li, Han Mei, Fang Chen, Qing Tang, Zhaoqing Yu, Xiaojian Cao, Binda T. Andongma, Shan-Ho Chou, Jin He

**Affiliations:** ^1^State Key Laboratory of Agricultural Microbiology, College of Life Science and Technology, Huazhong Agricultural University, Wuhan, China; ^2^Institute of Biochemistry and NCHU Agricultural Biotechnology Center, National Chung Hsing University, Taichung, Taiwan

**Keywords:** *Mycobacterium smegmatis*, transcriptome, transcriptional start site, leaderless mRNA, gene structural re-annotation, operon, sub-operon, highly active promoter

## Abstract

The non-pathogenic bacterium *Mycobacterium smegmatis* mc^2^155 has been widely used as a model organism in mycobacterial research, yet a detailed study about its transcription landscape remains to be established. Here we report the transcriptome, expression profiles and transcriptional structures through growth-phase-dependent RNA sequencing (RNA-seq) as well as other related experiments. We found: (1) 2,139 transcriptional start sites (TSSs) in the genome-wide scale, of which eight samples were randomly selected and further verified by 5′-RACE; (2) 2,233 independent monocistronic or polycistronic mRNAs in the transcriptome within the operon/sub-operon structures which are classified into five groups; (3) 47.50% (1016/2139) genes were transcribed into leaderless mRNAs, with the TSSs of 41.3% (883/2139) mRNAs overlapping with the first base of the annotated start codon. Initial amino acids of MSMEG_4921 and MSMEG_6422 proteins were identified by Edman degradation, indicating the presence of distinctive widespread leaderless features in *M. smegmatis* mc^2^155. (4) 150 genes with potentially wrong structural annotation, of which 124 proposed genes have been corrected; (5) eight highly active promoters, with their activities further determined by β-galactosidase assays. These data integrated the transcriptional landscape to genome information of model organism mc^2^155 and lay a solid foundation for further works in *Mycobacterium*.

## Introduction

*Mycobacterium tuberculosis* is the pathogen responsible for tuberculosis, which is second only to HIV/AIDS as the greatest cause of death worldwide. In fact, in 2015, 10.4 million people suffered from tuberculosis with 1.8 million deaths from the disease (World Health Organization WHO, 2016). The slow-growing and pathogenic features make it difficult to study on *M. tuberculosis*. Being a “fast grower” and non-pathogen, *M. smegmatis* has been used as a research model for *M. tuberculosis* to investigate on a wide variety of mycobacterial physiological processes, including drug resistance, dormancy, fatty acid metabolism, gene regulatory networks (Morbidoni et al., 2006; Cordone et al., 2011; Yang et al., 2012; Deng et al., 2015; Liu et al., 2016).

RNA-seq has recently emerged as a method allowing people to study RNA-based regulation in a genome-wide manner. Compared to microarray, RNA-seq exhibits unique advantages including: higher sensitivity, higher throughput, larger dynamic range, better genome sequence independence, and in single-nucleotide resolution (Wang et al., 2009), which make it more ideal for comprehensive systematic and accurate transcriptome analyses. There are two types of RNA-seq experiments, namely typical RNA-seq and differential RNA-seq (dRNA-seq) experiments. In a typical RNA-seq experiment, RNAs are converted to a cDNA library and amplified by PCR. Following deep sequencing, the sequences obtained are mapped to a reference genome for further data analysis. In the dRNA-seq library method, the original RNA sample is first treated with 5′-monophosphate-RNAs (5′-p-RNAs) terminator exonuclease (TEX) to specifically degrades 5′-p-RNAs but retain 5′-triphosphate-RNAs (5′-ppp-RNAs), thus relatively enriching the primary transcripts (5′-ppp-RNAs) (Sharma and Vogel, 2014). This method has been widely used to identify the primary 5′-ends in various bacteria (Sharma et al., 2010; Wurtzel et al., 2010; Cortes et al., 2013; Thomason et al., 2015; Babski et al., 2016). Compared to dRNA-seq, typical RNA-seq can also be used to identify the 5′-ends of transcripts, but it cannot distinguish the 5′-ends of primary transcripts from the 5′-ends of processed transcripts. However, the typical RNA-seq has unique advantages in quantifying gene expression levels and identifying operon structures.

In prokaryotic genomes, the typical RNA-seq could be applied to: (1) uncover the global transcriptional profile in genome scale, identify differentially expressed genes (DEGs) and determine the differences in transcriptional levels between wild type and its mutants at distinct conditions (Wang et al., 2013b; Li et al., 2016; Hendrickson et al., 2017); (2) identify 5′-ends of transcript (Wang et al., 2013a; Liao et al., 2015); single-nucleotide resolution enables RNA-seq to globally identify the first nucleotide in 5′-ends. Previously, 5′-ends of transcript were generally identified by 5′-Rapid Amplification of cDNA Ends (5′-RACE), which is troublesome and costly; (3) correct gene structural annotation (Perkins et al., 2009); currently, the majority of sequenced bacterial genomes are annotated by an algorithm-based program which is automated but error-prone. The combination of transcriptome data with algorithmic predictions could greatly facilitate gene structural annotation. (4) discover new genes (Arnvig et al., 2011; Ignatov et al., 2013). Mapping RNA-seq data to genome makes it possible to identify new genes (especially small non-coding RNAs) in intergenic region, within a gene, as well as those in the negative strand; (5) define operon and sub-operon structures (Wang et al., 2013; Fortino et al., 2014). Some genes in prokaryotes are organized into operons, which are co-transcribed to generate polycistronic transcripts. Through transcriptome mapping, we can clearly identify whether genes are transcribed together. Moreover, in operon, some internal genes possess their own TSSs (a typical symbol of sub-operon), which can be also elucidated by RNA-seq.

For *Mycobacterium*, RNA-seq has also been applied. Wang et al. (2013) applied RNA-seq to depict the operon structures in *M. marinum* and Cortes et al. (2013) used dRNA-seq to globally identify TSSs in *M. tuberculosis*. In mc^2^155, Shell et al. (2015) revealed a high abundance of leaderless mRNAs and small proteins by the combination of RNA-seq and ribosome profiling data. Other studies mainly focused on the identification of DEGs in different conditions (Petridis et al., 2015; Li et al., 2016; Wu et al., 2016; Hillion et al., 2017). However, no comprehensive insights have been achieved regarding *M. smegmatis* yet.

In the present study, we explored temporal gene expression profiles, identified 2139 TSSs throughout the genome, depicted 2233 operon and/or sub-operon structures, revealed a high proportion of leaderless mRNAs that were further verified them by Edman degradation, corrected structural annotation of 124 genes, and screened eight highly active promoters by combining growth-phase-dependent RNA-seq with related experiments in mc^2^155. Together, we have comprehensively examined the expression profiles and transcriptional structures of mc^2^155, which shall greatly facilitate further mycobacteria researches.

## Materials and methods

### Bacterial strains and growth conditions

Strains used in this study are listed in Table 1. *Escherichia coli* strain DH5a was grown in lysogeny broth (LB) medium. mc^2^155 wild type strain and its derivatives were grown at 37°C in Middlebrook 7H9 medium (Difco Becton Dickinson, USA) supplemented with 0.5% (v/v) glycerol and 0.05% (v/v) Tween 80, or on Middlebrook 7H10 agar (Difco Becton Dickinson, USA) supplemented with 0.5% (v/v) glycerol (Tang et al., 2014). When necessary, antibiotics were added to the culture containing either 50 μg/mL of kanamycin or 100 μg/mL of ampicillin.

**Table 1:** Strains used in this study.

**Strains**	**Characteristics or purposes**	**References**
mc^2^155	Wild-type *M. smegmatis* mc^2^155	Li and He, 2012
mc^2^155/pMV261-P*_*MSMEG*_0538_-lacZ*	P*_*MSMEG*_0538_* activity determination	This study
mc^2^155/pMV261-P*_*MSMEG*_0559_-lacZ*	P*_*MSMEG*_0559_* activity determination	This study
mc^2^155/pMV261-P*_*MSMEG*_0965_-lacZ*	P*_*MSMEG*_0965_* activity determination	This study
mc^2^155/pMV261-P*_*MSMEG*_1060_-lacZ*	P*_*MSMEG*_1060_* activity determination	This study
mc^2^155/pMV261-P*_*MSMEG*_1076_-lacZ*	P*_*MSMEG*_1076_* activity determination	This study
mc^2^155/pMV261-P*_*MSMEG*_1771_-lacZ*	P*_*MSMEG*_1771_* activity determination	This study
mc^2^155/pMV261-P*_*MSMEG*_2389_-lacZ*	P*_*MSMEG*_2389_* activity determination	This study
mc^2^155/pMV261-P*_*MSMEG*_2756_-lacZ*	P*_*MSMEG*_2756_* activity determination	This study
mc^2^155/pMV261-P*_*MSMEG*_3022_-lacZ*	P*_*MSMEG*_3022_* activity determination	This study
mc^2^155/pMV261-P*_*MSMEG*_3050_-lacZ*	P*_*MSMEG*_3050_* activity determination	This study
mc^2^155/pMV261-P*_*MSMEG*_3084_-lacZ*	P*_*MSMEG*_3084_* activity determination	This study
mc^2^155/pMV261-P*_*MSMEG*_3255_-lacZ*	P*_*MSMEG*_3255_* activity determination	This study
mc^2^155/pMV261-P*_*MSMEG*_3896_-lacZ*	P*_*MSMEG*_3896_* activity determination	This study
mc^2^155/pMV261-P*_*MSMEG*_4326_-lacZ*	P*_*MSMEG*_4326_* activity determination	This study
mc^2^155/pMV261-P*_*MSMEG*_4625_-lacZ*	P*_*MSMEG*_4625_* activity determination	This study
mc^2^155/pMV261-P*_*MSMEG*_4891_-lacZ*	P*_*MSMEG*_4891_* activity determination	This study
mc^2^155/pMV261-P*_*MSMEG*_4993_-lacZ*	P*_*MSMEG*_4993_* activity determination	This study
mc^2^155/pMV261-P*_*MSMEG*_5081_-lacZ*	P*_*MSMEG*_5081_* activity determination	This study
mc^2^155/pMV261-P*_*MSMEG*_5543_-lacZ*	P*_*MSMEG*_5543_* activity determination	This study
mc^2^155/pMV261-P*_*MSMEG*_5789_-lacZ*	P*_*MSMEG*_5789_* activity determination	This study
mc^2^155/pMV261-P*_*MSMEG*_6427_-lacZ*	P*_*MSMEG*_6427_* activity determination	This study
mc^2^155/pMV261-P*_*MSMEG*_6467_-lacZ*	P*_*MSMEG*_6467_* activity determination	This study
mc^2^155/pMV261-*MSMEG_4921*	Edman sequencing	This study
mc^2^155/pMV261-*MSMEG_6422*	Edman sequencing	This study
*Escherichia coli* DH5α	Cloning host	Stored by our laboratory
DH5α/pMD19-*MSMEG_2196*	Identification the TSS of *MSMEG_2196*	This study
DH5α/pMD19-*MSMEG_3756*	Identification the TSS of *MSMEG_3756*	This study
DH5α/pMD19-*MSMEG_3757*	Identification the TSS of *MSMEG_3757*	This study
DH5α/pMD19-*MSMEG_4985*	Identification the TSS of *MSMEG_4985*	This study
DH5α/pMD19-*MSMEG_5223*	Identification the TSS of *MSMEG_5223*	This study
DH5α/pMD19-*MSMEG_6235*	Identification the TSS of *MSMEG_6235*	This study
DH5α/pMD19-*MSMEG_6917*	Identification the TSS of *MSMEG_6917*	This study
DH5α/pMD19-*MSMEG_6920*	Identification the TSS of *MSMEG_6920*	This study

### RNA isolation and RNA-seq

mc^2^155 cells equivalent to 30 OD_600_ (e.g., 30 mL of 1 OD_600_ of one culture) were harvested from each sample (three growth phases each in two biological replicates) and then quickly-frozen in liquid nitrogen. The bacteria pellet was transferred into a mortar containing liquid nitrogen, and was lysed by constant grinding (in the presence of liquid nitrogen), into a fine powder. The powder was transferred to a 2 mL tube containing 1 mL TRIzol Reagent (Invitrogen, CA, USA), and shaken vigorously, until the powder dissolved uniformly. Then, 200 μL of chloroform was added to the mixture and mixed vigorously for 15 s. The mixture was incubated on ice for 5 min, followed by centrifugation at 10,000 × g for 15 min at 4°C. Equal volumes (400 μL) of upper clear supernatant and isopropanol were mixed in a fresh 1.5 mL tube to precipitate RNA. It was mixed immediately by gently inverting 8~10 times, and stored on ice for 15 min, followed by centrifugation at 10,000 × g for 30 min at 4°C. The upper clear phase was discarded, and 1 mL of 75% ethanol was added to wash the RNA pellet. The sample was gently washed by inverting 4–6 times, followed by centrifugation at 5,000 × g for 2 min at 4°C. Ethanol washing was repeated twice. Following the wash steps, ethanol was discarded. RNA was eventually dried by exposing to air for 5 min (air-dry) at room temperature. Finally, the RNA pellet was dissolved in 50 μL of RNase-free water, and stored at −80°C. Note that all reagents and materials used in this experiment were RNase-free.

For RNA-seq, 10 μg of total RNA from each sample was first treated with RNase-free DNase I (Takara, Japan) to prevent the potential contamination of genomic DNA. Ribosomal RNAs were removed using the RiboZero rRNA removal kit (Epicentre, USA) for gram-positive organisms prior to sequencing analysis. Hundered nanograms of rRNA-depleted RNA from each sample was fragmented into 200–300 nts and used as a template for randomly primed PCR. Strand-specific cDNA libraries were prepared by standard techniques for subsequent Illumina sequencing using the mRNA-seq Sample Prep kit (Illumina, USA). The resulting cDNAs were sequenced on an Illumina HiSeq 2500 (Shao et al., 2015).

### RNA-seq data analysis

The quality of the raw sequence data was first assessed by FastQC, the sequences further filtered and trimmed by Trimmomatic (Bolger et al., 2014). Clean reads were then used to map to the reference genome of mc^2^155 (NC_008596.1) by Bowtie2 (Langmead and Salzberg, 2012). BEDTools (Quinlan and Hall, 2010) analysis was performed on the alignments generated by Bowtie2 in order to quantify the mapped reads. To facilitate the comparison of expression levels of different samples, reads for CDSs were normalized with the total read count per sample and presented in the form of Reads Per Kilobase Per Million Reads (RPKM). The quantity of differential expressions of all transcripts was obtained using DEGseq (Wang et al., 2010) to include fold change values. In context, DEGs are those with two-fold changes or more, with FDR (false discovery rate) <0.001. As for single-nucleotide resolution transcriptome map, cleaned reads were mapped to mc^2^155 genome by BLASTN to get their genome location. Then Perl script was used to count the expression level of each nucleotide. Data were visualized using Artemis software.

### TSSs validation by 5′-RACE

To verify the TSSs identified by RNA-seq, 5′-RACE was performed using a tobacco acid pyrophosphatase (TAP)-based 5′-RACE kit according to manufacturer's instructions as previously described (Ali et al., 2017). The selected regions were amplified from 5′-RACE cDNA library using adapter primers (outer and inner primers) and gene specific primers (Table S1). After multiplex amplification, PCR products were purified and ligated into pMD19-T vector, and then transformed into *E. coli* DH5α (Table 1). Clones were sequenced and the first nucleotides next to 5′-RACE adaptor were considered to be the TSSs.

### Edman degradation to identify N-terminal amino acids

N-terminal sequencing was performed on proteins translated from leaderless mRNAs to identify their translation initiation amino acid residues. To get the test proteins, leaderless genes fused with 6 × His tag were over-expressed under the control of their own promoters based on the expression vector pMV261, and then, respectively, transformed into mc^2^155 strain to obtain a series of derivative strains. The native promoter could hardly be induced, so two highly expressed leaderless genes *MSMEG_4921* and *MSMEG_6422* were selected. For protein purification, the two mc^2^155 derivatives were harvested at mid-exponential phase, followed by cell lysis using French press. The clear lysate was loaded onto a Ni^2+^-NTA affinity column for protein purification. Purified proteins were applied on a 15% SDS-PAGE and then transferred to PVDF blotting membrane for Edman sequencing using PPSQ-31A protein sequencer (Shimadzu, Japan). The strains and primers used are listed in Table 1 and Table S1, respectively.

### Operon prediction

Operons prediction was based on the transcriptome map, visualized in Artemis. The prediction was carried out according to previously described method (Wang et al., 2013b). Briefly, genes in an operon share the same orientation, and transcripts should cover the intergenic region of the two adjacent genes. In addition, the ratio of the two gene expression levels should be <2. According to these two principles, we manually inspected the transcription of all 6,947 genes across the whole genome.

### β-galactosidase assay

β-galactosidase activity was performed in mc^2^155 by translational/transcriptional fusion of promoter to *lacZ* gene based on the expression vector pMV261. For translational fusion, the promoter regions, 5′-UTRs and partial N-terminal amino acids were fused with *lacZ* gene, while promoter and 5′-UTRs only were included in transcriptional fusion. P*hsp60* promoter was used as control when assessing the relative β-galactosidase activity of selected promoters (Yang et al., 2012). Reporter plasmids were transformed into wild type strain to obtain corresponding recombinant strains. All strains were grown in 7H9 medium at 37°C. β-galactosidase activity was determined as previously described (Miller, 1972). The strains and primers used are listed in Table 1 and Table S1, respectively.

## Results

### Global view of RNA-seq data at different growth phases

**Figure 1:**
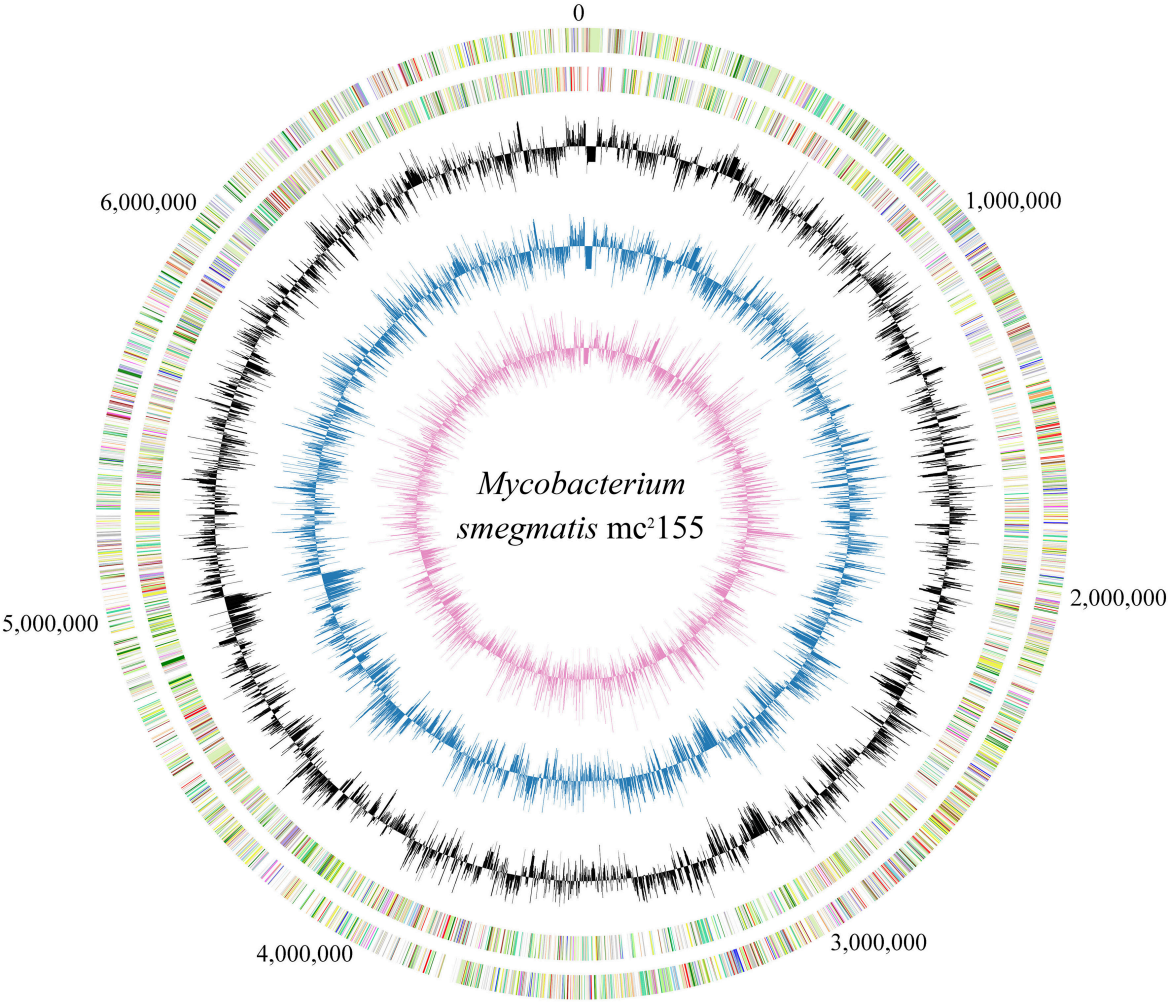
Circular map of *M. smegmatis* mc^2^155 genome and the corresponding transcriptome. Numbers outside circle show the genome coordinate. Moving inward, the subsequent two rings show CDSs in forward and reverse strands, respectively, with colors representing different COG categories. The inner three rings were colored in black, blue and purple representing the transcriptome maps at 16, 26, and 39 h, respectively; the uneven lines above and below the middle circle lines represent the expression level greater or lower than average.

The growth curve of mc^2^155 in 7H9 broth is a prerequisite for its transcriptome profile which comprises a series of growth-phase-dependent RNA-seq experiments. Results indicate that mc^2^155 reached mid-exponential phase at 16 h; early-stationary phase at 26 h and mid-stationary phase at 39 h (Figure S1). Two biological replicates were harvested from each of these three time points, and their total RNAs were extracted for subsequent experiments. All of the six RNA samples obtained from three different growth phases (two biological replicates) were found to exhibit high integrity with RNA Integrity Numbers (RINs) more than 9.5. Strand-specific cDNA libraries were then constructed and sequenced on an Illumina HiSeq 2000 platform. After removing low quality reads, 10 million clean reads were obtained for each library. The complete genome and trancriptome information of mc^2^155 are shown in Figure 1.

### Gene expression profiles during different growth phases

**Figure 2:**
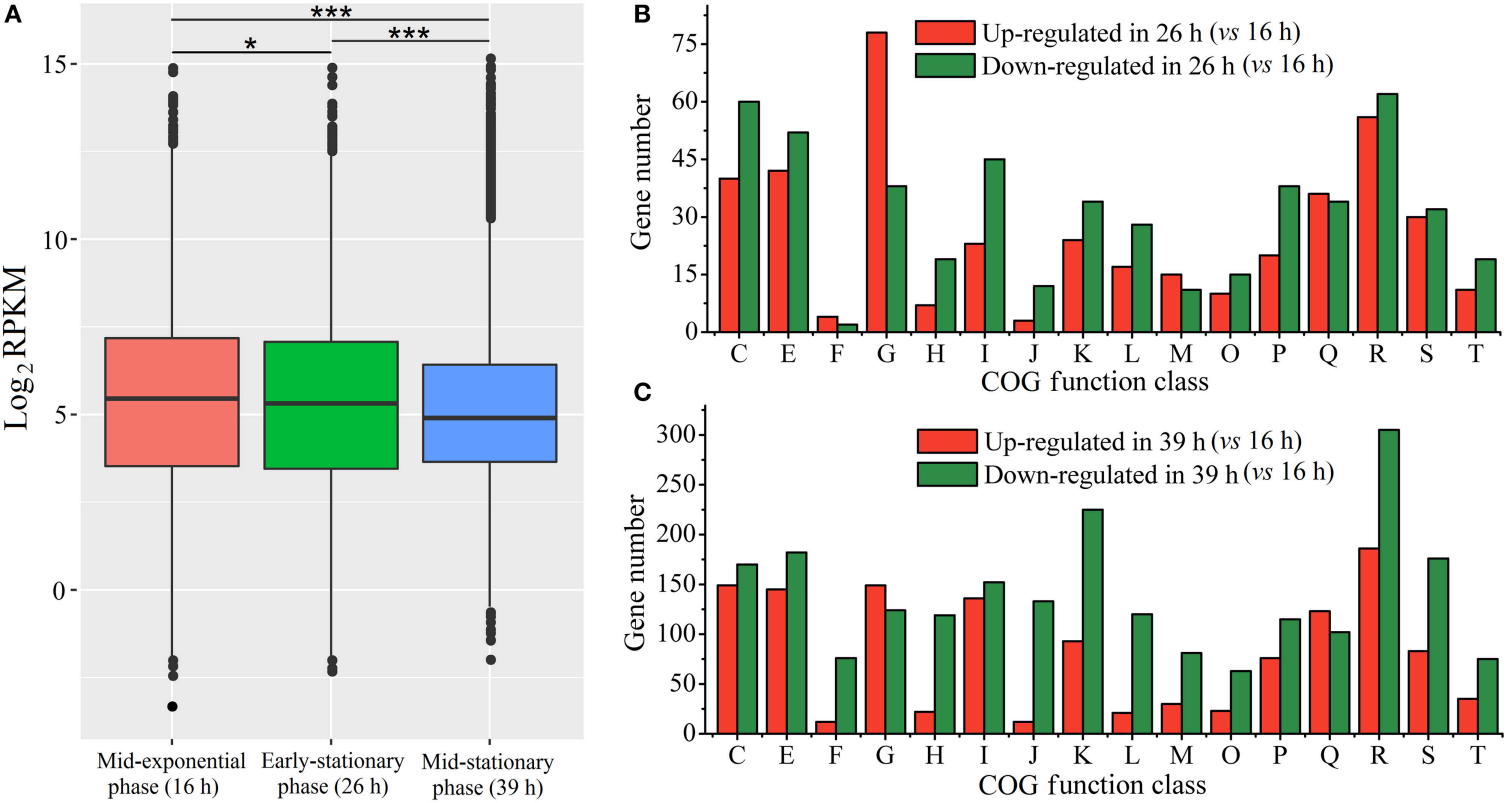
Gene expression levels in RNA-seq data. **(A)** The median expression levels of different growth phases measured by RPKM for whole identified genes. RPKM values of each sample were analyzed using Wilcoxon test, ^*^p < 0.05, ^**^p < 0.01, ^***^p < 0.001. **(B,C)** respectively indicate the number of DEGs in distinct COG functional categories at the early-stationary phase (26 h) and mid-stationary phase (39 h). The COG functional categories are as follows: C, energy production and conversion; E, amino acid transport and metabolism; F, nucleotide transport and metabolism; G, carbohydrate transport and metabolism; H, coenzyme transport and metabolism; I, lipid transport and metabolism; J, translation, ribosomal structure and biogenesis; K, transcription; L, replication, recombination and repair; M, cell wall/membrane/envelope biogenesis; O, posttranslational modification, protein turnover, chaperones; P, inorganic ion transport and metabolism; Q, secondary metabolites biosynthesis, transport and catabolism; R, general function prediction only; S, function unknown; T, signal transduction mechanisms.

In order to temporally investigate distinct biological processes, we analyzed gene expression profiles of the growth-phase-dependent RNA-seq data (Table S2). Comparison of RPKM values for each growth phase revealed significantly lower transcriptional levels for mid-stationary phase genes than those in the other two growth phases (Wilcoxon test, *p* < 0.001; Figure 2A). The differences in gene expression levels among the three growth phases were also analyzed with respect to different Cluster of Orthologous Groups of proteins (COG). DEGs were analyzed using mid-exponential phase RNA-seq data as control. In the mid-stationary phase, DEGs (especially down-regulated genes) are obviously increased in some clusters when compared to early-stationary phase (Figures 2B,C). We propose that in a cluster when the number of down regulated genes is far more than up-regulated genes, the cluster would be considered as down-regulated and *vice versa*. Thus, in the early-stationary phase (26 h), “carbohydrate transport and metabolism [G]” cluster was up-regulated, whereas “coenzyme transport and metabolism [H]”, “lipid transport and metabolism [I]” and “translation, ribosomal structure, and biogenesis [J]” clusters were down-regulated (Figure 2B). In the mid-stationary phase, “nucleotide transport and metabolism [F]”, “coenzyme transport and metabolism [H]”, “translation, ribosomal structure and biogenesis [J]”, and “replication, recombination and repair [L]” clusters were down-regulated; however, no cluster was up-regulated (Figure 2C).

**Figure 3:**
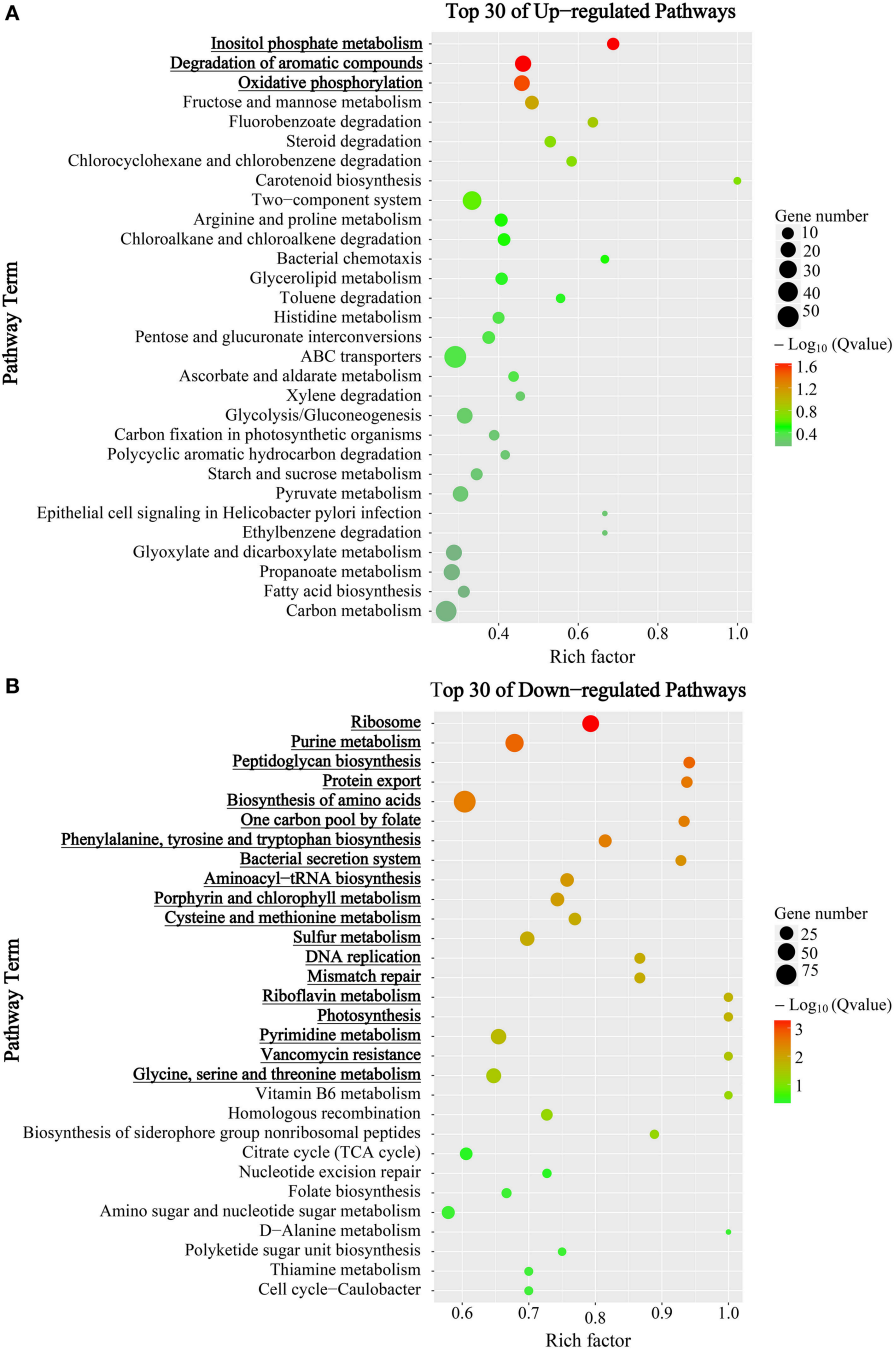
KEGG enrichment analysis of DEGs between mid-exponential and mid-stationary phases. Y-axis label represents the distinct KEGG pathways, and X-axis label represents rich factor (rich factor = amount of DEGs in the pathway/amount of all genes in background gene set). The colors of the dots represent the Q-values of enrichment. Red color indicates high enrichment, while blue color indicates low enrichment. Pathway terms were sorted by Q-value in ascending order; and were marked in bold and underlined when Q-value < 0.05. The sizes of the dots represent the gene number of enrichment. **(A)** Top 30 up-regulated KEGG pathways. **(B)** Top 30 down-regulated KEGG pathways.

Furthermore, DEGs were subjected to Kyoto Encyclopedia of Genes and Genomes (KEGG) pathways analysis to find out temporally associated biological functions. As shown in Figure 3, pathways with member genes significantly regulated (Q-value < 0.05) were marked in bold and underlined. As mentioned above, transcriptional levels of most genes were substantially reduced in mid-stationary phase; however, most of the genes in “inositol phosphate metabolism”, “degradation of aromatic compounds” and “oxidative phosphorylation” pathways were up-regulated (Figure 3A and Figure S2). In contrast, the majority of genes were significantly down-regulated, especially genes in “translation” (including “ribosome” and “aminoacyl-tRNA biosynthesis” pathways) process (Figure 3B and Figure S2).

### Genome-wide identification of TSSs

**Figure 4:**
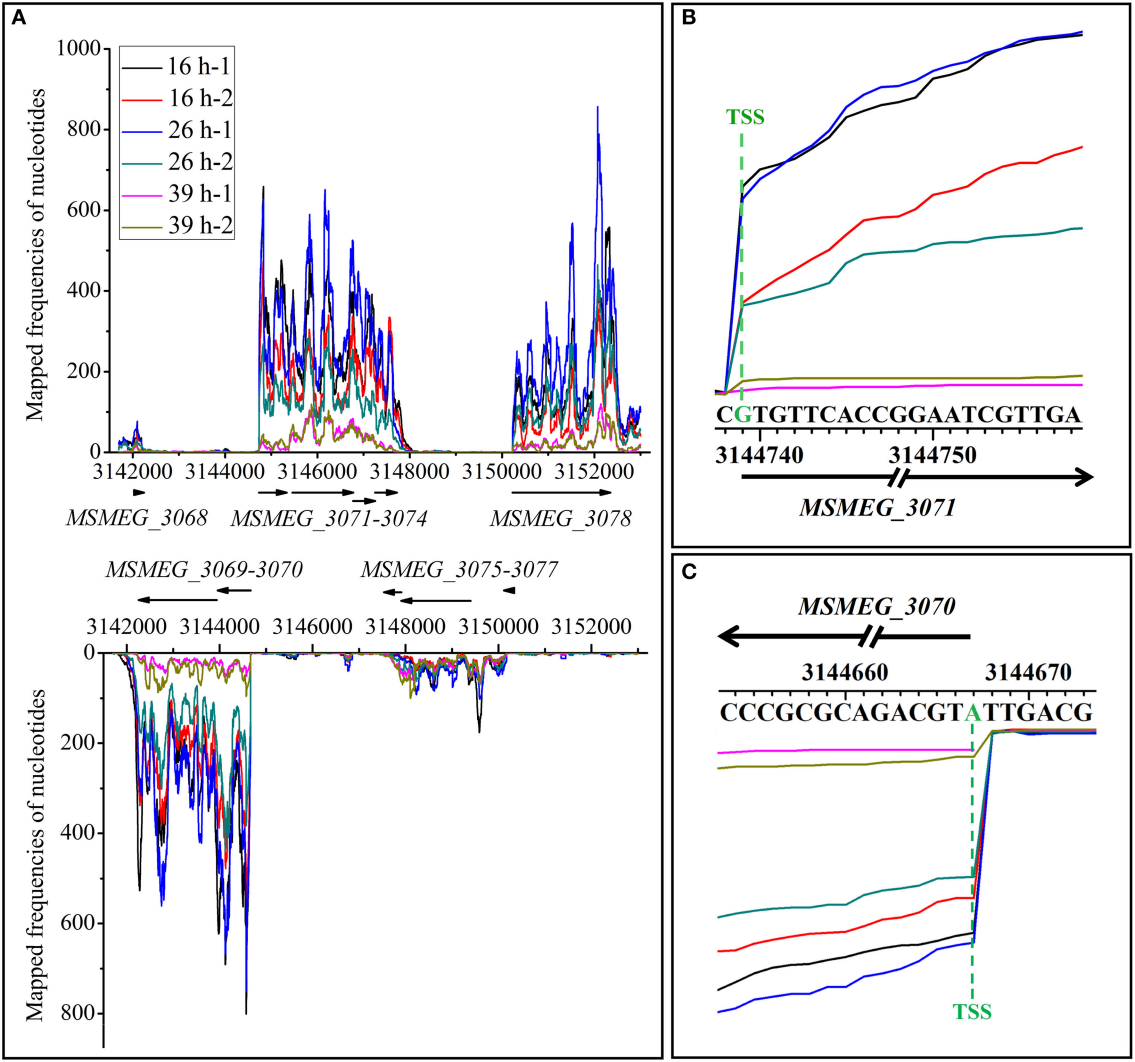
Transcriptional maps of *MSMEG_3068-3078* loci. **(A)** Whole view of strand-specific transcriptional maps of *MSMEG_3068-3078* loci. *MSMEG_3068, MSMEG_3071-3074*, and *MSMEG_3078* genes residing in the positive strand while genes of *MSMEG_3069-3070* and *MSMEG_3075-3077* are located in the negative strand. **(B)** Single-nucleotide resolution transcriptional maps of *MSMEG_3071* and its TSS. **(C)** Single-nucleotide resolution transcriptional maps of *MSMEG_3070* and its TSS. Lines of different colors represent samples in different growth phases, −1 and −2 represent the two biological replicates. The same as below.

Transcriptional start sites (TSSs) typically refer to the 5′-ends of primary transcripts, but not 5′-ends of the processed transcripts. In our transcriptome data, most of the 5′-ends of transcripts (except for those of processed transcripts) are indeed their TSSs, thus we used the term “TSSs” in the following text. To locate TSSs in genome-wide scale, clean reads were mapped to mc^2^155 genome to generate single-nucleotide resolution transcriptome maps. However, the uneven transcriptome map caused by sequencing makes it hard to identify all the TSSs, especially internal TSSs which could typically imply the existence of small RNAs. Therefore, to improve the accuracy and reliability of our study, we only focused on the TSSs found at the beginning of mRNA transcripts. We manually inspected the transcriptome maps separately and successively. As shown in Figure 4A, the whole view of strand-specific transcriptional maps of *MSMEG_3068*-*3078* gene cluster was uneven, which is common in RNA-seq caused by sequencing bias. The first nucleotide significantly enriched at the beginning of transcript is considered to be the TSS. Single-nucleotide resolution of transcriptional maps showed that the transcriptions of *MSMEG_3071* and *MSMEG_3070* genes were clearly enriched at TSSs (Figures 4B,C). In this way, we totally identified 2139 TSSs throughout the mc^2^155 genome (Table S3). To further confirm TSSs identified by RNA-seq, eight transcripts were randomly selected and verified by the canonical 5′-RACE method (Table S4). The results showed that TSSs identified by RNA-seq and 5′-RACE experiments overlapped at an identical nucleotides, which validates the accuracy of RNA-seq based TSSs identification. In general, promoters are located immediately upstream of TSSs. Thus, sequences 50 nts upstream of identified TSSs were scanned for potential conserved promoter motif by MEME. As a result, a conserved −10 motif (TANNNT) was found ranging 7–12 bp upstream of the identified TSSs in 69.5% of the genes, but no conserved −35 motif could be identified. These findings are similar to a previous study in *M. tuberculosis* (Cortes et al., 2013).

**Figure 5:**
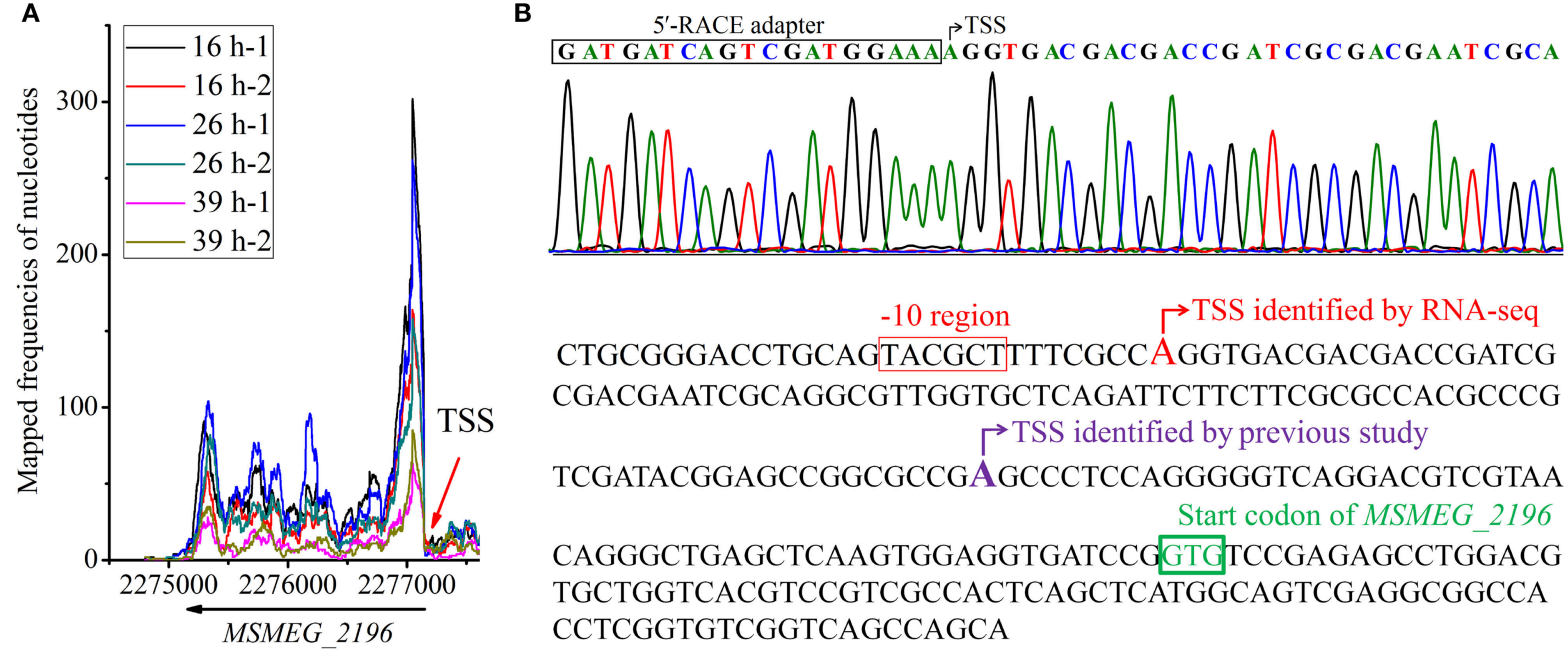
Identification of *MSMEG_2196* TSS. **(A)** Whole view of transcriptional maps of *MSMEG_2196-2199* loci. **(B)** TSS of *MSMEG_2196* identified by 5′-RACE in this study. **(C)** Comparison of TSS of *MSMEG_2196* identified by our RNA-seq data with that previously reported (Bharati et al., 2013). The new identified TSS is colored in red, and the putative −10 motif is marked in rectangle. The previously identified TSS is colored in purple, however, no conserved −10 motif can be found.

*MSMEG_2196* is a c-di-GMP synthetase encoding gene, the TSS of which was first determined in a previous study (Bharati et al., 2013). In our study, its TSS was detected by RNA-seq and was validated by 5′-RACE (Figure 5). Interestingly, the reported TSS is 88 nts downstream of our identified location. Moreover, a conserved −10 motif could be found upstream of our identified TSS but not in the previous one, indicating that our study could provide more credible data for gene organization. Taken together, our systematic identification of TSSs in genome scale could facilitates the research of gene expression regulation in mc^2^155.

### High proportion of leaderless mRNA in mc^2^155

**Figure 6:**
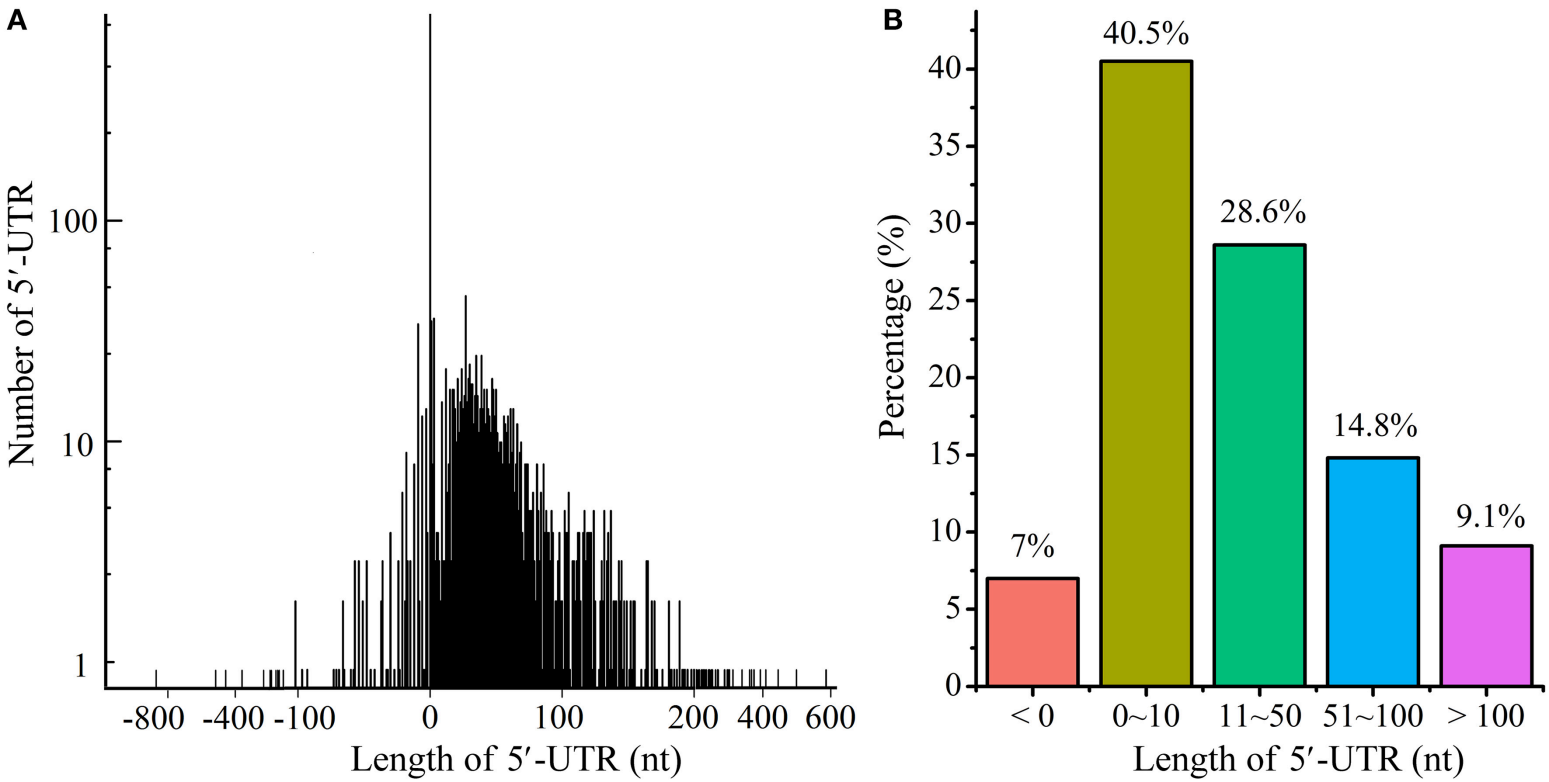
Distribution of 5′-UTRs length. **(A)** The distribution of 5′-UTRs length. **(B)** The percentage of 5′-UTRs length. The length of 5′-UTRs <0 indicated potentially mis-annotated genes.

Based on TSS identification, we systematically explored the 5′-UTR length throughout the transcriptome (Figure 6A and Table S3). As shown in Figure 6B, 40.5% of the 5′-UTRs were <10 nts in length, and were considered to be leaderless mRNA; 28.6% of transcripts have 5′-UTR length from 11 to 50 nts, and 14.8% from 51 to 100 nts. In addition, 9.1% of transcripts exhibited long 5′-UTRs (with length more than 100 nts). Previous study indicated that long 5′-UTRs might hide regulatory elements such as riboswitch and transcription-attenuation region, which would regulate the transcription of downstream genes in a more sophisticated way (Breaker, 2011; Wang et al., 2013a; Rosinski-Chupin et al., 2014; Dersch et al., 2017). It is worth mentioning that about 7% TSSs were identified downstream of the annotated start codon, which were rather unlikely and were therefore considered to be mis-annotated.

**Figure 7:**
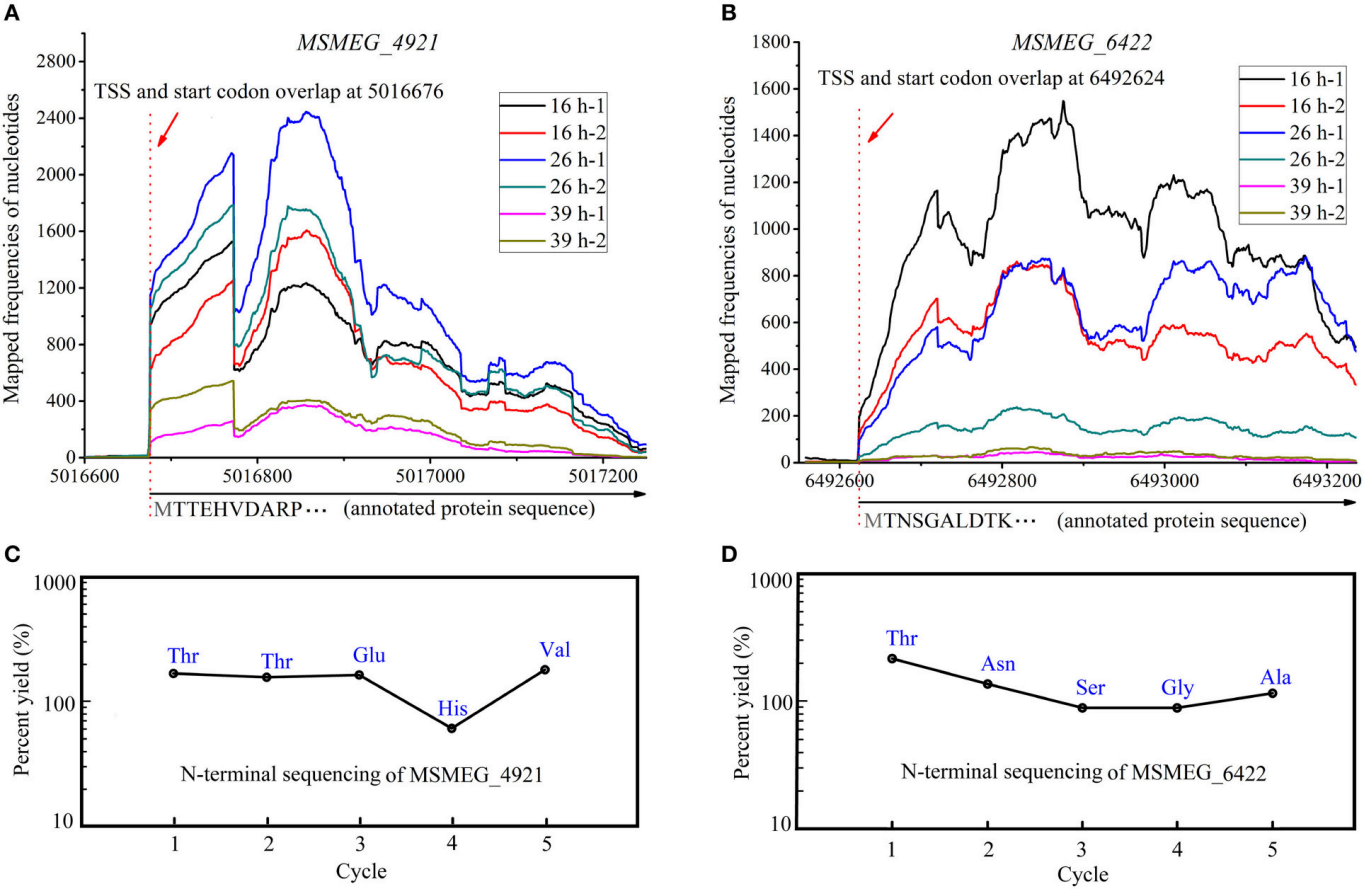
N-terminal amino acids identification of MSMEG_4921 and MSMEG_6422. **(A)** and **(B)** respectively represent the transcriptional maps of *MSMEG_4921* and *MSMEG_6422*, while **(C)** and **(D)** respectively show the N-terminal five amino acids of MSMEG_4921 and MSMEG_6422 proteins. Both amino acid sequences are identical to the annotation files. Noteworthy, the N-terminal translation initiator Met was removed by methionine amino peptidase (MetAP), which is often crucial for the function and stability of proteins.

We have observed that ~40.5% (866/2139) of the transcripts with annotated TSSs had a 5′-UTR <10 nts in length, likely generating leaderless mRNAs. Moreover, the TSSs of 35.48% (759/2139) mRNAs overlapped with the first nucleotide of annotated start codon (AUG/GUG). In bacteria, the Shine-Dalgarno (SD) sequence helps to recruit the ribosome to the mRNA to initiate translation. One would ponder how ribosome binds to mRNA to start the translation procedure without an SD sequence. Can these mRNA be efficiently translated? Do these widespread leaderless mRNAs use other specific translation mechanisms or are they simply mis-annotated? Therefore, it is important to confirm the translation of leaderless mRNA and identify the initial amino acid of the translated protein. Shell et al. (2015) tried to reveal the widespread translation of leaderless mRNA in mc^2^155 by ribosome profiling; however, the result could only indicate that ribosome did bind to the leaderless mRNAs but could not directly verify the translation of leaderless mRNAs. Moreover, they had also performed a proteomics study (Orbitrap LC-MS) on *M. tuberculosis* H37Rv to confirm this feature. As a result, a large number of proteins translated from leaderless mRNAs were identified. However, the key point in the validation of leaderless feature is to determine whether the first N-terminal amino acid of the translated protein is in accordance with the initial nucleotides of leaderless mRNA. LC-MS cannot be used to efficiently identify the initial amino acid though some peptides detected in that study were identical to the annotated start codons. Thus, we performed Edman degradation, which is the most credible method in initial amino acid identification, on the MSMEG_4921 and MSMEG_6422 proteins both translated from leaderless mRNAs (Figures 7A,B). To get the test protein, leaderless genes fused with 6 × His tag were over-expressed under the control of their own promoters based on the expression vector pMV261, and were respectively transformed into mc^2^155 strain to obtain a series of derivative strains. Purified proteins were applied on a 15% SDS-PAGE and then transferred to PVDF blotting membrane for Edman sequencing. The results indicated that their five N-terminal amino acids were identical to those annotated in the genome (Figures 7C,D). This strongly confirmed the leaderless feature of these leaderless mRNAs. Noteworthy, the N-terminal translation initiator Met was removed by methionine amino peptidase (MetAP), which is often crucial for the function and stability of proteins. Collectively, leaderless mRNA seems to be the common feature of mycobacteria. Further studies are required to understand how these leaderless mRNAs could be successfully translated into proteins. Several studies have shown that the unique translation can be initiated with high efficiency at leaderless transcripts via the undissociated 70S/80S ribosome and the initiator tRNA (Moll et al., 2004; Udagawa et al., 2004; Andreev et al., 2006; Giliberti et al., 2012). However, it is unclear whether mycobacterial strains employ this mechanism to initiate the translation of the high-proportion leaderless mRNA.

### Correction of mis-annotated genes in mc^2^155

**Figure 8:**
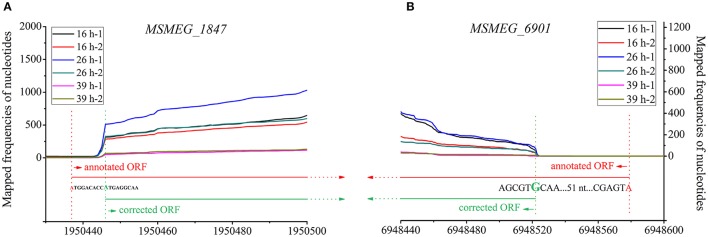
Transcriptional maps and gene structural re-annotation of *MSMEG_1874* **(A)** and *MSMEG_6901* **(B)**. The nucleotides in red and red dot lines indicate the start codons annotated by algorithms-based software. The nucleotides in green and green dot lines indicate the TSS determined by RNA-seq, and these TSSs are also considered to be the accurate start codons of relevant genes.

The majority of sequenced bacterial genomes were annotated by algorithm-based software, which can be error-prone. As shown in Figure 6, the lengths of 5′-UTRs of 150 transcripts were negative, indicating that these corresponding genes were very likely mis-annotated in these assay conditions (three different growth phases in 7H9 medium). Further explorations found that 124 out of these 150 transcripts used AUG/GUG as the first three nucleotides at TSS (Table S5), which were similar to the leaderless mRNAs. Given the fact that leaderless mRNAs are widespread in mc^2^155, and an AUG/GUG at TSS is sufficient for the initial translation of leaderless mRNA in mc^2^155 (Shell et al., 2015), we therefore propose that in these assay conditions, the 124 mis-annotated genes were transcribed into leaderless mRNA. Furthermore, distances between the identified TSSs and the first nucleotide of annotated start codons were in multiples of three in length, indicating that the corrected gene structural annotation does not shift the original open reading frame. Figure 8 schematically shows the transcriptional maps of two mis-annotated proteins *MSMEG_1874* and *MSMEG_6901*, as well as the corrected versions. Together, we propose that RNA-seq could be highly useful to correct gene mis-annotation.

### Genome-wide identification of operons and sub-operons

Some genes in prokaryotes are organized into operons, which are then transcribed into polycistronic transcripts. It is typically considered that genes in an operon are functionally related. Zhang and He (2013) reported that overexpression of *MSMEG_6080* in mc^2^155 led to cell expansion and aggregation; however, co-overexpression of *MSMEG_6080* and *MSMEG_6078*, located in a similar operon, alleviated the phenotypes described above instead. Furthermore, a gene in the middle of an operon, which can be independently transcribed in response to certain stimuli, is called sub-operon. Therefore, identification of genes that are grouped together into operons/sub-operons is important to studies of gene function and complex regulatory networks.

**Figure 9:**
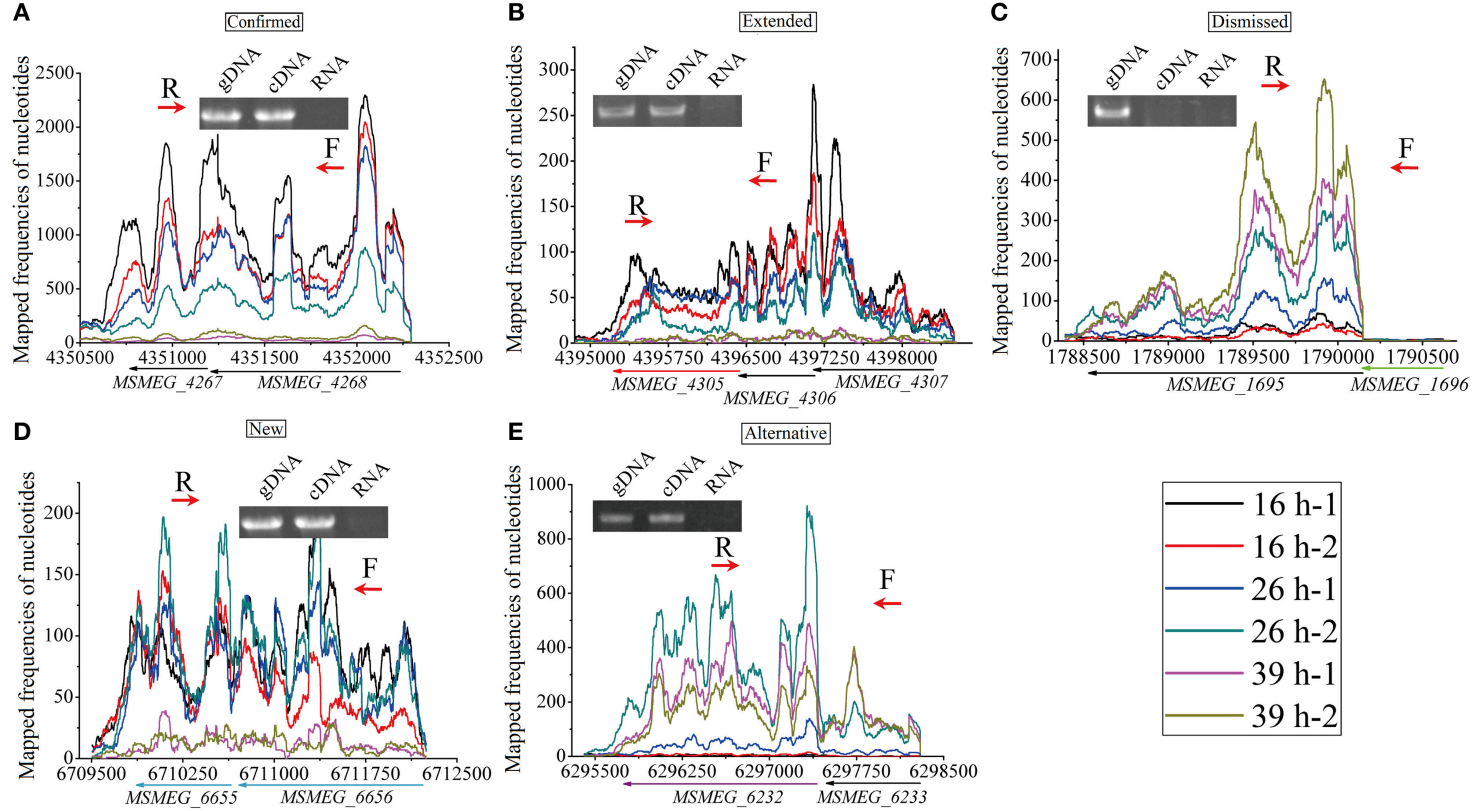
Examples of the five groups of identified operon. The RT-PCR forward (F) and reverse (R) primers are indicated in red arrows. **(A)** Confirmed, DOOR annotated *MSMEG_4268* and *MSMEG_4267* as an operon, our RNA-seq data and RT-PCR experiment also indicate this. **(B)** Extended, DOOR annotated *MSMEG_4307* and *MSMEG_4306* as an operon, without *MSMEG_4305* (in red arrow); however, our RNA-seq data and RT-PCR experiment found that the transcription was extended to *MSMEG_4305*. **(C)** Dismissed, DOOR annotated *MSMEG_1696* to *MSMEG_1695* (in green arrow) as an operon, however, our RNA-seq data and RT-PCR experiment indicated that *MSMEG_1696* was dismissed from transcription. **(D)** New, our RNA-seq data identified new operons not found by DOOR, which was also indicated by RT-PCR. **(E)** Alternative, *MSMEG_6233* and *MSMEG_6232* (in purple arrow) were found to be co-transcribed; and *MSMEG_6232* seems to be alternatively transcribed from its own TSS.

To investigate the structures of operons and sub-operons in genome scale, we manually inspected the transcription of all 6,947 genes across the genome, and finally identified 2,233 operons/sub-operons (Table S6). The 2,233 operons/sub-operons were further classified into five groups, according to the prediction of operons by DOOR (Mao et al., 2014). These five groups are: (1) confirmed, which means that our results were consistent with those predicted by DOOR (Figure 9A); (2) extended, in which we found that more genes could be transcribed into the polycistronic mRNA (Figure 9B); (3) dismissed, where less genes were found to be transcribed together (Figure 9C); (4) new, where some genes were transcribed together but were not annotated by DOOR (Figure 9D); and (5) alternative, indicating the existence of sub-operon (Figure 9E). To further illustrate the reliability of our results, several operons of each group were randomly selected for reverse transcription PCR (RT-PCR), and the results were consistent with RNA-seq data. The selected operons/sub-operons in each group is shown in Table 2.

**Table 2:** Operon prediction in *M. smegmatis* mc^2^155.

**Operon groups**	**Total number**	**Number of genes selected for RT-PCR**	**Genes selected for RT-PCR^f^**
Confirmed^a^	1635	7	*MSMEG_1556-1557*, *MSMEG_3255-3254*, *MSMEG_3540-3542*, *MSMEG_3569-3567*, *MSMEG_3655-3656*, *MSMEG_3689-3688*, *MSMEG_3935-3933*
Extended^b^	61	7	*MSMEG_1812-1811*, *MSMEG_1810-1809*, *MSMEG_3024-3025*, *MSMEG_3503-3504*, *MSMEG_3794-3793*, *MSMEG_3887-3996*, *MSMEG_4087-4085*
Dismissed^c^	167	6	*MSMEG_1525-1527*, *MSMEG_1873-1874*, *MSMEG_1951-1952*, *MSMEG_2279-2280*, *MSMEG_2356-2357*, *MSMEG_3104-3105*
New^d^	65	5	*MSMEG_1402-1403*, *MSMEG_3499-3450*, *MSMEG_3616-3615*, *MSMEG_3685-3686*, *MSMEG_3942-3941*
Alternative^e^	273	2	*MSMEG_3946-3945*, *MSMEG_6234-6535*

a Confirmed: in an operon, RNA-seq annotated equal number of gene to DOOR;

b Extended: in a operon, RNA-seq annotated more genes than DOOR;

c Dismissed: in a operon, RNA-seq annotated less genes than DOOR;

d New: this operon was not annotated by DOOR;

e Alternative: sub-operon, a gene inside of an operon was transcribed independently;

f *of each groups, several genes were selected to perform RT-PCR*.

### Screening and identification of highly active promoter

As mentioned above, mc^2^155 is widely accepted as a model organism in mycobacterial researches and has usually been used to overexpress homologous proteins from *M. tuberculosis* and *M. bovis* due to its non-pathogenic and fast-growing features (Soares et al., 2014; Deng et al., 2016). The temperature-sensitive P*hsp60* promoter could induce the expression of downstream genes on heat shock conditions, typically via a temperature shift from 37 to 45°C (Batoni et al., 1998). The inducibility makes P*hsp60* the most widely used promoter in mycobacteria. However, there are also some problems existing in the inducible expression system. Therefore, more promoters need to be discovered for driving gene expression in mycobacteria.

**Figure 10:**
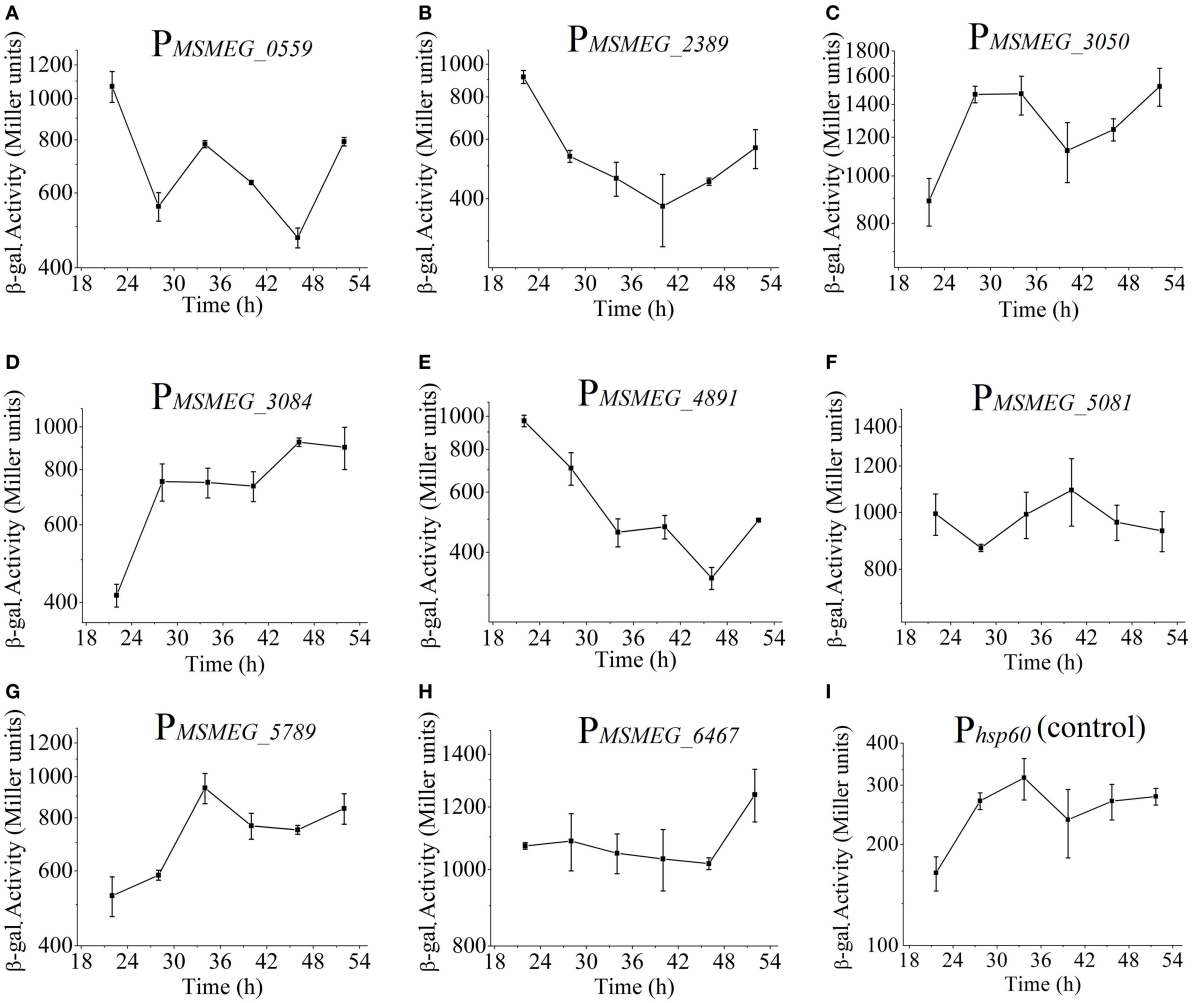
Screening and identification of highly active promoter. P_*MSMEG_0559*_ represents mc^2^155/pMV261-P_*MSMEG*_*0559*_*-lacZ* strain, and the same as below. β-galactosidase activities for the nine strains (*Phsp60* as a control) were shown in **(A–I)**. Data represent the averages of biological triplicates. Error bars indicate standard deviation.

It is widely known that many non-coding RNAs such as rRNA, tRNA, 6S RNA, possess a highly active promoter. Here, we intended to screen alternate highly active promoters for gene expression. According to RNA-seq data, 22 gene candidates with high RPKM values were selected (Table S7). By fusing these promoter sequences to *lacZ* gene, we successfully certified that 8 out of 22 candidates exhibiting high β-galactosidase activities that are 3–5 times higher than the control promoter P*hsp60* (Figure 10). Additionally, some promoters showed temporal characteristics. As shown in Figures 10C,D, the promoter activities of P_*MSMEG*_3050_ and P_*MSMEG*_3084_ increased with time. In contrast, P_*MSMEG*_4891_ only exhibited high promoter activities in the early growth phase (Figure 10E). However, P_*MSMEG*_5081_ showed a relatively constant expression level during the whole detection period (Figure 10F). It is noteworthy that the β-galactosidase activity assays do not correlate well with RNA-seq data. This may be due to the fact that RNA-seq data only denote the transcriptional level. However, β-galactosidase activity assays reflect the expression levels of both transcription and translation. The disagreement between mRNA and protein levels usually happens, since many modifications may occur either at post-transcriptional or translational levels (McCarthy and Gualerzi, 1990; Alifano et al., 1994; Kozak, 2005).

## Discussion

This study combined RNA-seq with experimental evidences to generate a global overview of the transcriptional landscape of mc^2^155, including genome-wide annotations of the transcriptional structures, as well as a bunch of non-canonical TSSs, 5′-UTRs and operons/sub-operons. For the first time, we have also performed Edman degradation to validate the translation of leaderless mRNA. In addition, eight highly active promoters were discovered for gene overexpression in mycobacteria.

In order to reveal the comprehensive transcriptome of mc^2^155, we carried out RNA-seq experiments at three different growth phases. As expected, most of genes showed a lower expression level in mid-stationary phase, especially genes related to replication, translation, and DNA repair biological processes. However, the majority of genes in “inositol phosphate metabolism” and “degradation of aromatic compounds” pathways were found to be significantly up-regulated in the mid-stationary phase. Being noteworthy is that as a soil bacterium, mc^2^155 is similar to other soil mycobacterial isolates such as *Mycobacterium* KMS, MCS, and JLS that share organic compounds degradation capability. Almost all genes in the “degradation of aromatic compounds” pathway exhibit increasing expression level in a temporal order (Figure 3A and Figure S2). This might indicate increased utilization of aromatic compounds as carbon source in mc^2^155 during the stationary phase, demonstrating the potential of mc^2^155 as an efficient pollutant eliminator.

Compared to other bacteria, mc^2^155 seems to contain a strikingly high proportion (46.06%) of leaderless mRNA. Furthermore, for 40.99% of the TSSs identified in our study, the TSS overlapped with the first nucleotide of the start codon. Previously, Cortes et al. (2013) reported that 26% of TSSs overlapped with annotated start codon in *M. tuberculosis* H37Rv. Transcriptomics research in *M. avium* also revealed a high proportion (33%) of leaderless mRNAs (Ignatov et al., 2013). Furthermore, the translations of leaderless mRNAs were confirmed by Edman degradation in this study. Therefore, the high proportion of leaderless mRNAs may be a common feature of mycobacteria. In fact, Zheng et al. (2011) provided some insights into genes encoding leaderless mRNA in prokaryotes by a bioinformatics method, revealing that leaderless mRNA are widely spread in a variety of bacteria, especially in phyla *Actinobacteria* and *Deinococcus*-*Thermus*. Moreover, previous experimental studies have also revealed a high proportion of leaderless mRNA in some prokaryotes especially in Archaea (Torarinsson et al., 2005; Babski et al., 2016; Bauer et al., 2017).

It has long been known that the 5′-UTR of bacterial mRNAs include specific sequence elements for guiding ribosome binding, i.e., the SD motifs that interact directly with complementary motifs (anti-SD motifs) in the 16S rRNA. However, the initiation of leaderless mRNA translation is poorly understood. Currently, there are two possible pathways for initiation of leaderless mRNA translation in bacteria (verified in *E. coli*). One is mediated by the formation of the 30S–fMet-${\text{tRNA}}_{\text{f}}^{\text{Met}}$-IF2 complex (Grill et al., 2000). With the help of initiation factor IF2, Met-tRNA^Met^ can bind to the ribosome 30S subunit, and the ternary complex (30S–fMet-${\text{tRNA}}_{\text{f}}^{\text{Met}}$-IF2) then recognizes the start codon of leaderless mRNA to initiate translation. The other mechanism is mediated by 70S ribosomes (Moll et al., 2004; Udagawa et al., 2004). The non-dissociated 70S ribosome first combines with fMet-tRNA to recognize and bind the start codon (AUG or GUG) of leaderless mRNA to initiate translation. Importantly, both mechanisms have been found in *E. coli* that possesses a low proportion of leaderless mRNA with weak translation capability. However, robust translation of leaderless mRNA was validated by β-galactosidase reporter system in mycobacteria (Shell et al., 2015). Moreover, by analyzing the proteome data of mc^2^155 (Chopra et al., 2014), we found that the levels of protein translated from mRNAs with or without leader sequence were almost equal. Thus, leaderless mRNAs seem to be rather abundant and are robustly translated in mycobacteria when compared to *E. coli*. Yet, the two leaderless mRNA translation pathways currently present in *E. coli* (possessing low proportion of leaderless mRNA and weak translation capability) may not be sufficient to explain the existence of high proportion of leaderless mRNA and the strong translation capability in mycobacteria. There may exist a plethora of uncharacterized mechanisms for ribosomal recognition and translational initiation of leaderless mRNAs in mycobacteria.

Bharati et al. (2013) reported that *MSMEG_2196*, a diguanylate cyclase encoded gene, could either be co-transcribed into a polycistronic mRNA (*MSMEG_2199*-*MSMEG_2196*) or independently as a monocistronic mRNA. Similarly, the independent transcription of CH1330 as a malate dehydrogenase gene in an operon in *Bacillus thurgiensis* CT-43 has been demonstrated (Wang et al., 2013b). It is now commonly believed that sub-operons are regulated by specific transcriptional factors and sigma factors in response to different stresses. Meanwhile, this could also be an efficient strategy for prokaryotes since they are usually composed of relatively smaller genomes. As mentioned above, we classified the identified operons into five groups, which comprise 273 sub-operons. We suppose that some of the sub-operons found here could exhibit alternate functions in mc^2^155, which require further efforts to investigate.

Being widely used as a model organism in mycobacterial researches, mc^2^155 should be capable of expressing and over-expressing genes from many other homologous mycobacteria. Currently, the P*hsp60* promoter is the only one that is widely used for expression of mycobacterial genes because of its heat-induced characteristic. However, there are some problems existing in this inducible expression system. Firstly, proteins produced at high temperature usually fail to fold into native structures and result in a mis-folded or inactive state. Moreover, high temperature induction may exhibit a disadvantage in introducing DNA mutations, which may disable or alter target gene expression (Al-Zarouni and Dale, 2002; Buddle et al., 2002). Additionally, there are also serious doubts about the stability of plasmids carrying the P*hsp60* promoter (Haeseleer, 1994; Al-Zarouni and Dale, 2002). Therefore, our discovery of eight highly active and temporarily-expressed promoters could contribute significantly to the gene expression in mc^2^155.

Noteworthy, we found that some promoters exhibited great differences in β-galactosidase activities when fused translationally to *lacZ* gene compared to those fused transcriptionally. Translational fusion of some promoters exhibited β-galactosidase activity about 5–10 times higher than that of transcriptional fusion. For translational fusion, the promoter regions, 5′-UTRs and part of the N-terminal amino acids encoding regions were fused together to the *lacZ* gene. However, only promoter and 5′-UTRs were included in the transcriptional fusion. The CDS near the start codon may be important for translation of some genes.

## Data accession number

RNA-seq data have been submitted to GEO under the accession number GSE103158.

## Author contributions

XL performed most of the experiments and made most of the data evaluation. HM completed most of the bioinformatics analysis. JH and HM gave the main idea of this research. FC, QT, ZY, and XC participated in partial experiments and interpretation of the data. XL, HM, and JH conceived the study and drafted the manuscript. JH, S-HC, and BA revised the manuscript. All authors read and approved the final manuscript.

### Conflict of interest statement

The authors declare that the research was conducted in the absence of any commercial or financial relationships that could be construed as a potential conflict of interest.

## Supplementary material

Figure S1 Growth curve of mc^2^155. mc^2^155 was cultured in 250 mL Erlenmeyer containing 100 mL 7H9 medium at 37°C, under a rotary shaker at 200 r/min. Overnight cultured mc^2^155 was used to inoculate fresh media at a starting OD_600_ of 0.02, and OD_600_ was determined by sampling cultures in every 3 h. Samples for RNA-seq at 16, 26, and 39 h, corresponding to mid-exponential, early-stationary and mid-stationary phases respectively, were collected, with two biological replicates.

Figure S2 Heatmap of all genes in the three pathways. Heatmap in each picture were measured by Log_2_ (fold_change) of selected genes. **(A)** Degradation of aromatic compounds pathway. **(B)**Translation process. **(C)** TInositol phosphate metabolism.

Table S1 Primers used in this study.

Table S2 Gene expression profiles of *M. smegmatis* mc^2^155.

Table S3 Genome-wide identification of TSSs in *M. smegmatis* mc^2^155.

Table S4 Eight genes randomly selected to perform 5′-RACE experiments.

Table S5 Structural re-annotation of genes in *M. smegamtis* mc^2^155.

Table S6 Genome-wide operon (sub-operon) identificaiton.

Table S7 Expression profiles of genes selected to screen high activity promoters.
