# Supplementary figures and images for: Transcriptome Landscape of Mycobacterium smegmatis
Source: Front Microbiol. 2017 Dec 18;8:2505. doi: 10.3389/fmicb.2017.02505 (PMC5741613; doi:10.3389/fmicb.2017.02505)

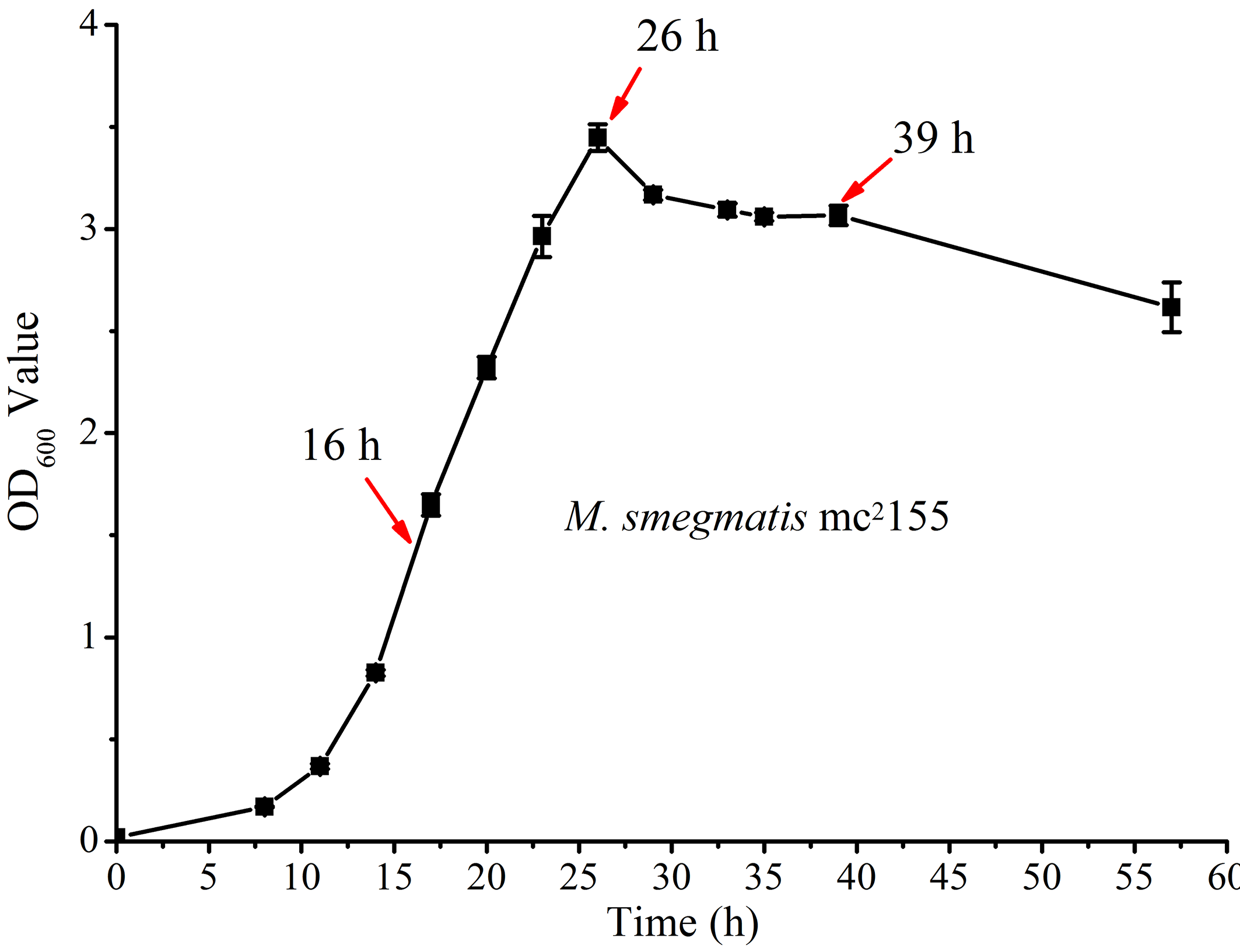

Supplement: Figure S1 — Growth curve of mc2155. mc2155 was cultured in 250 mL Erlenmeyer containing 100 mL 7H9 medium at 37°C, under a rotary shaker at 200 r/min. Overnight cultured mc2155 was used to inoculate fresh media at a starting OD600 of 0.02, and OD600 was determined by sampling cultures in every 3 h. Samples for RNA-seq at 16, 26, and 39 h, corresponding to mid-exponential, early-stationary and mid-stationary phases respectively, were collected, with two biological replicates. [file Image1.TIF]

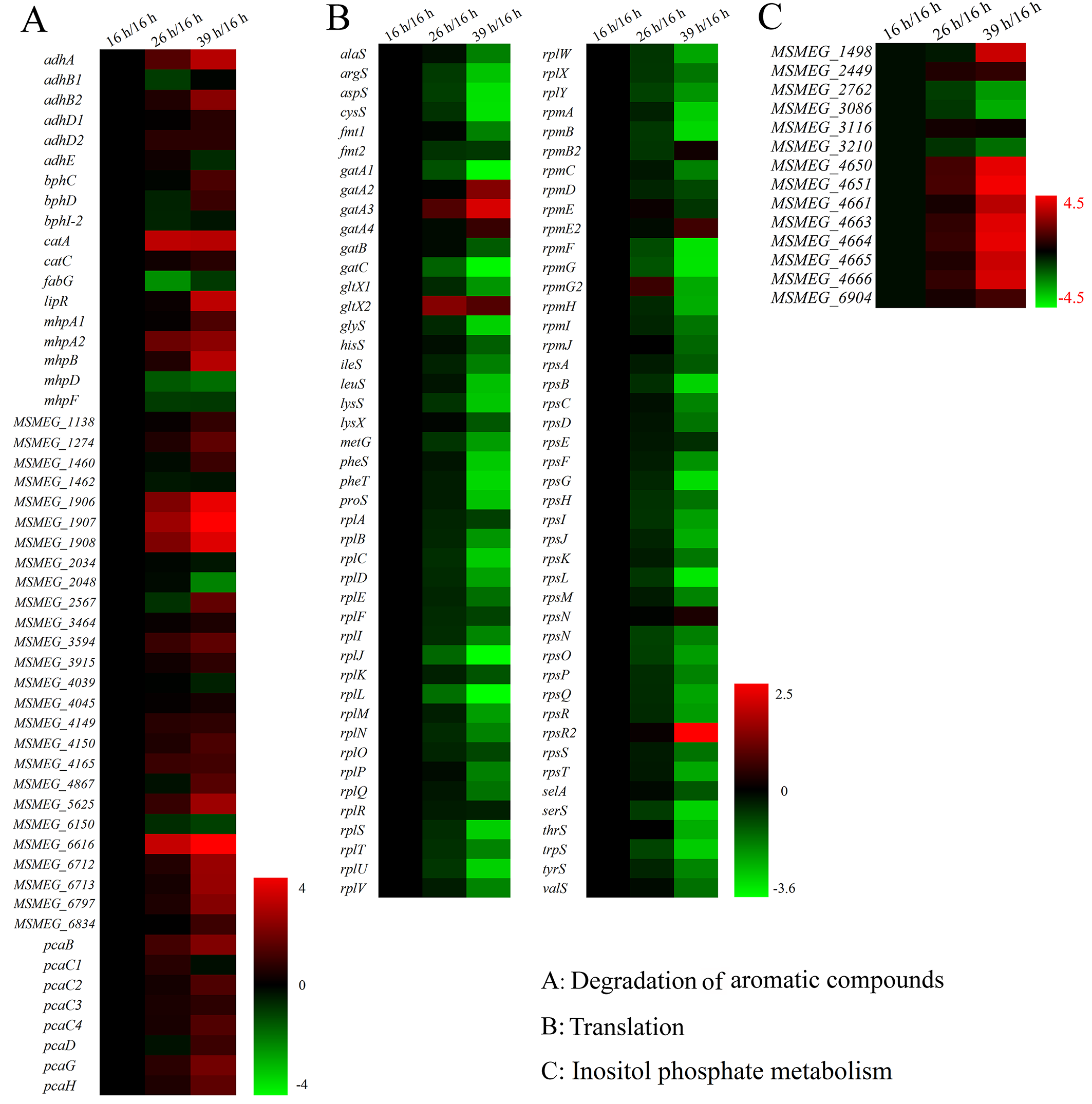

Supplement: Figure S2 — Heatmap of all genes in the three pathways. Heatmap in each picture were measured by Log2 (fold_change) of selected genes. (A) Degradation of aromatic compounds pathway. (B)Translation process. (C) TInositol phosphate metabolism. [file Image2.TIF]
